# Valley Fever: Pathogenesis and Evolving Treatment Options

**DOI:** 10.7759/cureus.50260

**Published:** 2023-12-10

**Authors:** Spencer C Zaheri, Elizabeth Field, Cody A Orvin, Dominique M Perilloux, Rachel J Klapper, Anitha Shelvan, Shahab Ahmadzadeh, Sahar Shekoohi, Alan D Kaye, Giustino Varrassi

**Affiliations:** 1 School of Medicine, Louisiana State University Health Sciences Center, Shreveport, USA; 2 Department of Radiology, Louisiana State University Health Sciences Center, Shreveport, USA; 3 Department of Anesthesiology, Louisiana State University Health Sciences Center, Shreveport, USA; 4 Department of Pain Medicine, Paolo Procacci Foundation, Rome, ITA

**Keywords:** antifungal, inhalation, arthroconidia, fungus, valley fever, coccidioidomycosis

## Abstract

Coccidioidomycosis, also termed Valley fever, is a fungal infection caused by the inhalation of *Coccidioides* endospores. Once inhaled by a human host, the arthroconidia endospores travel to the lungs' alveoli to transform into spherules that grow and rupture to release more endospores. In the host immune response, macrophages, neutrophils, and dendritic cells will recognize the fungal antigen, producing pro-inflammatory cytokine. Th2 lymphocytes (type 2 helper T cells) are theorized to be the main human defense against *Coccidioides *given that Th2 deficiency is seen in patients with disseminated forms of the disease. A common triad of symptoms of coccidioidomycosis, also called "desert rheumatism," include fever, erythema nodosum, and arthralgia, often accompanied by a respiratory problem. In a clinical setting, along with the evaluation of symptoms, a medical provider may also test the patient's blood using antibody tests or perform microscopy to directly detect the presence of *Coccidioides *in a patient tissue sample for confirmation of a diagnosis. Imaging modalities may also be used to determine lung involvement and assess disease progression. A majority of coccidioidomycosis cases do not require specific treatment and will resolve on their own, so an approach with symptomatic treatment in mind is appropriate. If symptoms do not resolve, azoles or amphotericin B may be used, with the standard drug of choice being fluconazole (Diflucan, Pfizer, New York, New York, United States). Treatment varies depending on the immunocompetency of the patient. To name a few, pregnant patients and those with history of human immunodeficiency virus (HIV) or transplantation require special considerations.

## Introduction and background

Valley fever, also known as coccidioidomycosis, is a dimorphic fungal infection caused by the inhalation of the spores of *Coccidioides immitis* and/or *C. posadasii*; the disease itself is localized to arid climates within the western hemisphere [1-3]. *Coccidioides* was first discovered in 1892 after fervent study of the disease in the southwestern region of the United States, but it was not properly identified until later [1,2]. One area worth noting in the study of the disease is the San Joaquin Valley in California, which was a location of heightened endemicity and from which the disease derived its namesake [1,2]. Other areas of the United States with heightened endemicity include southern Arizona, west Texas, and southern New Mexico [3]. Upon its discovery, *C. immitis* infection was considered fatal for three decades until a student exposed to the fungus in a laboratory recovered from the infection, prompting further testing and subsequently leading to the development of a *Coccidioides* skin test [2]. It was then discovered that many asymptomatic residents of the San Joaquin Valley were test-positive, along with the fact that many natives suffered from fevers, pneumonia, and erythema nodosum, showing the geographic prevalence of the disease and varying presentations [2]. Although coccidioidomycosis may be acquired through specific climates and geographic areas, its scale of impact is substantial when considering the increasing incidence and distribution of the disease, whether it be among natives or those traveling within endemic areas [2]. Coccidioidomycosis has many different clinical presentations ranging from asymptomatic to minor respiratory infection to chronic pulmonary disease. Because of these factors, diagnosis and treatment methods are complex.

## Review

Epidemiology

Coccidioidomycosis is a fungal infection caused by the inhalation of spores of the fungus *Coccidioides* [4]. The disease is endemic to certain regions in the Southwestern United States, particularly in the arid and semiarid areas of Arizona, California, Nevada, Utah, New Mexico, and Texas [4]. It is also found in areas of Mexico and Central and South America [4]. The incidence of coccidioidomycosis has been increasing over the past few decades, especially in the United States [4]. According to the Centers for Disease Control and Prevention (CDC), the annual incidence of reported coccidioidomycosis cases in the United States was 15.2 cases per 100,000 people in 2019, with 97% of cases coming from Arizona and California [5]. In comparison, the CDC reported 5.3 cases of coccidioidomycosis per 100,000 people in 1998, exemplifying an almost 300% increase in incidence [6].

Several factors contribute to the epidemiology of coccidioidomycosis, one being environmental conditions [4]. *Coccidioides* fungi thrive in arid and semiarid regions with alkaline soil, with outbreaks often occurring after periods of drought followed by rain [4]. Windstorms, earthquakes, and construction activities, among others, can also contribute to the dissemination of fungal spores, increasing the risk of exposure [4]. Another factor at play is population susceptibility. Individuals with certain risk factors, such as immunocompromised individuals (e.g., those with HIV/acquired immunodeficiency syndrome (AIDS) or receiving immunosuppressive therapy), pregnant women, incarcerated individuals, and certain racial or ethnic groups (e.g., African Americans, Filipinos, and Native Americans), are more likely to develop severe forms of coccidioidomycosis [4]. Transplant recipients previously infected with coccidioidomycosis are also at greater risk of reinfection post transplant [4]. There seems to be a male gender bias for the incidence of coccidioidomycosis; however, certain areas, like Arizona, have recently seen a rise in female cases [4].

Furthermore, older individuals are more at risk for developing Valley fever; specifically, people living in California between the ages of 40 and 49 and people in other endemic areas of the United States aged 65 or older have a greater likelihood of contracting coccidioidomycosis [4]. An individual's susceptibility to coccidioidomycosis infection can also be affected by their choice of occupation [4]. For example, people whose work exposes them to more airborne spores in soil, such as farmers and construction workers, are at higher risk to develop infection [4]. Surveillance and reporting of coccidioidomycosis cases are essential for understanding the epidemiology of the disease. Health departments and healthcare providers play a crucial role in monitoring and reporting cases to local and national authorities. Improved awareness and testing for coccidioidomycosis can lead to earlier diagnosis and appropriate management [7]. This is so important for controlling the spread of coccidioidomycosis because there are limited scientifically proven prevention methods. Currently, tactics being used in hopes of preventing Valley fever include wetting soil prior to digging, wearing masks, and spreading awareness [4].

Coccidioidomycosis, therefore, is an emerging fungal infection with a growing incidence in certain endemic regions. Environmental factors, population susceptibility, and improved surveillance contribute to our understanding of the disease's epidemiology. Further research is needed to better understand the factors influencing the increased incidence and to develop more effective prevention and control strategies [4].

Diagnosis

Valley fever may provide a challenging diagnosis to many due to its wide range of clinical presentations and the similarities of its symptoms with other respiratory illnesses [8]. The diagnosis of coccidioidomycosis typically involves a combination of clinical evaluation, laboratory tests, and imaging studies. During the clinical evaluation, healthcare providers take into account the patient's medical history, including travel to endemic areas and potential exposure to *Coccidioides* fungi [9]. They also assess the symptoms, which can vary from mild flu-like symptoms to severe pneumonia or disseminated disease [9]. Someone who suffers from a symptomatic pulmonary coccidioidomycosis infection may present with fever, chills, headache, joint pain, chest pain, coughing, difficulty breathing, weight loss, night sweats, and fatigue [9]. A common presentation of symptoms includes fever, erythema nodosum, and arthralgia; this triad is frequently referred to as "desert rheumatism" [9]. Someone who suffers from an extrapulmonary infection may have no respiratory symptoms and can pose a tricky diagnosis given how disseminated coccidioidomycosis can mimic many other conditions [9]. Certain populations, such as those with weakened immune systems, pregnant women, and specific racial or ethnic groups, are at a higher risk of developing severe forms of the disease. Occupational exposure and geographic location in endemic regions also contribute to the risk of coccidioidomycosis [9].

Laboratory tests play a crucial role in the diagnosis of coccidioidomycosis. The detection of *Coccidioides* antibodies in the blood is commonly used as a diagnostic tool, with the two main types of antibodies tested being immunoglobulin M (IgM) and immunoglobulin G (IgG) [8]. IgM antibodies are typically present during acute infection, while IgG antibodies indicate past or chronic infection. These antibody tests, such as enzyme immunoassays (EIAs), complement fixation, or immunodiffusion tests, can help confirm the diagnosis [8]. In certain cases, the direct detection of the *Coccidioides* fungus may be necessary. This can be done through methods such as microscopy, where samples from respiratory secretions, tissue specimens, or other bodily fluids are examined under a microscope with Papanicolaou, potassium hydroxide, and calcofluor for the presence of the characteristic fungal structures, specifically a spherule [8]. Fungal cultures can also be performed to isolate and identify the organism, but they can take several weeks to yield results [10]. Imaging studies, such as chest X-rays, magnetic resonance imaging (MRIs), or computed tomography (CT) scans, are useful in evaluating the extent of lung involvement and identifying complications, like lung nodules, cavities, or pleural effusions, as well as homing in on areas of the body specifically affected by the disease [9]. These imaging modalities can help guide the diagnosis and monitor disease progression [9]. Notably, the diagnosis of coccidioidomycosis may require a multidisciplinary approach, involving infectious disease specialists, pulmonologists, and laboratory professionals with experience in fungal diagnostics. Additionally, healthcare providers need to consider the clinical context, including the patient's symptoms, travel history, and risk factors, in order to make an accurate diagnosis [9]. False-negative and false-positive results may occur in the laboratory tests for coccidioidomycosis, highlighting the need for careful interpretation of the results in conjunction with the patient's clinical presentation [11]. False-negative results tend to occur more often in individuals who have other illnesses or medications that disrupt skin tests or people previously infected with Valley fever [10].

Therefore, the diagnosis of coccidioidomycosis involves a combination of clinical evaluation, laboratory tests to detect antibodies or the fungus itself, and imaging studies. Due to the varied clinical manifestations of the disease and the potential for diagnostic challenges, healthcare providers need to consider the patient's medical history, symptoms, and risk factors to make an accurate diagnosis. Collaboration between healthcare professionals and the use of appropriate diagnostic methods are critical for the early and accurate detection of coccidioidomycosis.

Pathogenesis

Coccidioidomycosis is mostly benign and self-limiting but may also be progressive and spread throughout the body [12]. When symptoms do arise due to a *Coccidioides* infection, they mimic influenza or pneumonia symptoms such as fever, chills, headache, severe joint pain, chest pains, and coughing [13]. Usually, the symptoms resolve spontaneously within a few weeks or months [12]. The more progressive form of a *Coccidioides* infection is referred to as disseminated coccidioidomycosis or coccidioidal granuloma and can present with nodules or cavities in the lung parenchyma, lymph node involvement, bone lesions, osteomyelitis, or meningitis, which is usually the immediate cause of death from disseminated coccidioidomycosis [13]. The granulomas that can form in the lungs from a chronic *Coccidioides* infection vary in size and distribution and are typically composed of epithelioid cells, multinucleated giant cells, and lymphocytes [14]. *Coccidioides* resides within dust and soil, where the species undergoes autolysis, a process in which some fungal cells are transformed into barrel-shaped, loosely adherent endospores called arthroconidia [1]. Slight soil disturbances from instances like construction, farming, or windstorms are the primary routes for the arthroconidia spores to become airborne and infiltrate their host through inhalation [1]. The length of the arthroconidia is only 2-5 microns, allowing them to reach the terminal bronchioles following inhalation [1]. Once in the lungs' alveoli, the human body temperature allows these spores to transform from a rectangular form into a more unique structure called spherules, also referred to as the parasitic form of *Coccidioides* [12]. In the spherule form, *Coccidioides* grow to the size of 75-100 microns in diameter due to the internal division of developing septae containing 100-300 endospores each [1]. Eventually, the growing spherules rupture, releasing the endospores, which are then recognized as fungal antigens by alveolar macrophages, neutrophils, and dendritic cells, leading to the production of pro-inflammatory cytokines such as interleukin-1 (IL-1), interleukin-6 (IL-6), interleukin-12 (IL-12), and tumor necrosis factor-alpha (TNF-α) [15]. These cytokines attract and activate other immune cells, initiating an acute and local inflammatory response and aid in the beginning of an adaptive immune response. However, as spherules grow larger, the innate effector cells become ineffective, including neutrophils, monocytes, and natural killer cells [1]. Additionally, when the spherules rupture and the endospores are released, each endospore can develop into new spherules if the host's immune system fails to repress the growth of the fungus [16]. The adaptive immune response gets involved via CD4+ T-helper cells differentiating into Th1 lymphocytes following IL-12 release from activated antigen-presenting cells (APCs) [15]. Once Th1 lymphocytes become activated, they begin secreting interferon-gamma (IFN-γ) to enhance macrophage activity [15]. However, it is theorized that Th2 lymphocytes are the primary defense against *Coccidioides* species, given that Th2 deficiency or dysfunction has been frequently observed in patients with extrapulmonary or disseminated forms of the disease [1].

Among mammals, coccidioidomycosis lesions are typically limited to the lungs and vary from focal lung lesions or multifocal [17]. A retrospective study that observed 79 cases of camelids diagnosed with coccidioidomycosis found that 66 (84%) of them had lung lesions resulting in pneumonia and 62 (78%) with multifocal liver lesions [17]. This study confirmed the identification of gross anatomic lesions of coccidioidomycosis being similar across all mammals and organs studied, but the extent and severity of the lesions varied greatly [17]. The gross observation of the pyogranulomatous lesions appeared yellow to white with central necrosis, ranged from multifocal to coalescing, were roughly and randomly distributed across each organ, and ranged in diameter from 0.2 cm to 10 cm surrounded by a rim of immune cells. In the retrospective camelid study, the target organs of the *Coccidioides* species identified a decreasing order of prevalence, starting with the lung, liver, lymph nodes, spleen and kidney, heart, skin, and then skeletal muscle [17]. This finding confirms that the respiratory route is the primary portal of entry for *Coccidioides* species across mammals affected.

Moreover, the increasing variability in the severity of coccidioidomycosis leads scientists to look into understanding the distribution of genotypes among the *Coccidioides* species populations and human host variations [16]. In 2010, Neafsey et al. successfully sequenced 10 genomes for the *C. immitis* and *C. posadasii* species [18]. This data confirmed the presence of hybridization and genetic introgression between species, thus suggesting each's participation in natural selection influencing *Coccidioides* biogeography and virulence factors between species [16]. Sexual structures have never been identified in either species of *Coccidioides*, but this genetic introgression suggests a sexual phase must exist [19]. In a population genetic study using polymerase chain reaction (PCR) amplification, screening for single-stranded conformation polymorphisms, and direct DNA sequencing, Burt et al. aimed to discover mating molecular markers at the nucleotide level and found the *C. immitis* undergo almost complete recombination, not clonal growth [19]. Then, a more directed search for mating or meiosis by Fraser et al. identified mating type alleles (MAT) in both *Coccidioides* species, typical alleles for heterothallic bipolar mating systems, and one idiomorph containing high-mobility-group (HMG) box characteristics defining the MAT1-2 allele and another containing alpha-box gene defining the MAT1-1 allele [20]. These findings give an explanation to the unique genotypes found across numerous strains of *Coccidioides* species, thus influencing the differing pathological findings in individuals with no identified immunocompromising defect [16].

The number of studies done to assess the human immune response to coccidioidomycosis is limited due to *Coccidioides* mainly being asymptomatic, however; as mentioned earlier, the studies on the human immune response all tend to suggest that cell-mediated immunity, part of the adaptive immune response, plays the most critical role [16]. The observation of past clinical reports of humans affected by coccidioidomycosis paints a clear picture of two groups: symptomatic patients with a specific defect in the human immune response or symptomatic patients showing higher susceptibility without a yet identified defect in the immune response [16]. Acute coccidioidomycosis is characterized by lung inflammation with numerous neutrophils, lymphocytes, and plasma cells and spherules filled with endospores. As the acute form progresses, necrosis and abscesses begin to form, leading to chronic infection, marked by lung fibrosis, scarring, and granulomas at various stages, some of which may contain calcifications [21]. Progressive lung fibrosis in patients ultimately results in respiratory impairment and reduced lung function [21]. In severe cases, meningitis can develop, causing neurological symptoms and potentially leading to long-term disabilities or death [22]. Overall, the prevalence and severity of coccidioidomycosis infections among mammals depend on species susceptibility, environmental conditions, level of exposure, comorbidities, and immunosuppressive states [17]. Even though the lungs are the primary organ affected, the infection can disseminate hematogenously to other organs. Pathological findings range from acute inflammation to chronic granulomatous reactions, and understanding these features of *Coccidioides* species is crucial for the accurate diagnosis, effective management, and prevention of potential complications of this fungal infection.

Treatment

Most cases of coccidioidomycosis do not require treatment or can simply be resolved via symptomatic treatment [23]. These symptomatic treatments can include nonsteroidal anti-inflammatory drugs (NSAIDs) as well as rest. If symptoms do not subside, there are two classes of drugs generally used for treatment: azoles and amphotericin B. Azoles work by inhibiting the synthesis of ergosterol in the cell membrane [24]. There are two different categories of azoles: imidazoles and triazoles. Imidazole use is generally limited to the treatment of superficial mycoses [25]. Triazoles, however, are considered safer because of their greater affinity for the cytochrome P-450 enzyme and thus have broader uses [25]. Both fluconazole and itraconazole are triazoles. The current preferred drug to treat coccidioidomycosis is fluconazole (Diflucan, Pfizer, New York, New York, United States) [1]. The recommended dosage of fluconazole is between 400 mg and 1200 mg daily [1]. Possible side effects of fluconazole and itraconazole include hair loss, dry skin, and dry lips [23]. These side effects are typically resolved after treatment has been halted. A second medication that is used for the treatment of coccidioidomycosis is amphotericin B. This drug is the drug of choice in severe or refractory cases of coccidioidomycosis [24]. Amphotericin B utilizes sterols in the cell membrane to cause intracellular leakage, ultimately resulting in cell death [24]. Possible complications and side effects of amphotericin B include headaches, paresthesia, myelopathy, and sensation loss [24]. For this reason, amphotericin B is only used in severe or refractory cases. At this time, fluconazole and itraconazole have proven to be safer than amphotericin B [25]. Recent studies show that corticosteroids can be used to hasten recovery from a coccidioidomycosis infection. In the past, corticosteroids have not been used during the treatment of coccidioidomycosis because of the fear of increasing the virulence of the fungus, but the effectiveness of corticosteroid treatment has been questioned due to its limited efficacy [26]. Corticosteroids can be used as an adjunctive therapy to treat acute respiratory distress syndrome that may arise due to coccidioidomycosis [26]. Treatment of immunocompromised hosts living in an endemic area may differ from that of immunocompetent hosts. For patients with HIV infection, all patients receive an antifungal therapy consisting of either fluconazole or itraconazole [27]. Patients on drugs associated with immune suppression have an increased risk of acquiring a coccidioidomycosis infection. These patients should discontinue the medication that is causing the suppressed immune system in order to begin antifungal treatment and may eventually start the immunosuppressant again following antifungal treatment [27]. In the case of transplant recipients, all patients that live in an endemic area should be screened prior to transplantation. Those with a history of coccidioidomycosis should receive 200 mg of fluconazole daily for a duration of six months. If a patient has a positive serologic test, 400 mg of fluconazole is recommended daily for the duration of a year followed by lifelong suppressive therapy in conjunction with 200 mg of fluconazole daily [27]. Lastly, special considerations must be taken for pregnant patients. Women that acquire coccidioidomycosis during their second or third trimester should take a triazole such as fluconazole as treatment [27]. Azoles should not be used during the first trimester of pregnancy as they are teratogenic at that time (Table 1) [27].

**Table 1:** Drug treatment of Valley fever (coccidioidomycosis)

Drug	Dosage and route of administration	Adverse effects	Contraindications	Mechanism of action
Fluconazole [28]	Daily doses of 600-1000 mg of fluconazole orally	Gastrointestinal symptoms: nausea, abdominal pain, vomiting, and diarrhea. Other symptoms: anaphylaxis, hepatotoxicity, asthenia, myalgia, fever, leukopenia, and insomnia	Fluconazole should be avoided in patients with hypersensitivity to fluconazole or any portions of its formula. Avoid co-administration of fluconazole with QT-prolonging drugs. Fluconazole is contraindicated in patients with hereditary fructose malabsorption as sucrose is an ingredient in oral fluconazole.	Fluconazole interacts with a cytochrome P-450 enzyme to inhibit the production of ergosterol in the fungal cell membrane. This increases cellular permeability.
Itraconazole [29]	200 mg orally every 12 hours	Rare adverse effect: cardiotoxicity. More common adverse effects: hepatotoxicity and gastrointestinal disturbances (diarrhea, vomiting, nausea)	The main contraindication is heart failure due to itraconazole's cardiotoxic effects. A second contraindication is liver failure due to its metabolism by cytochrome P-450. Lastly, it is contraindicated in pregnant women as it is teratogenic.	Itraconazole inhibits ergosterol synthesis, thus destabilizing the fungal cell wall. Itraconazole is metabolized by the cytochrome P-450 enzyme.
Amphotericin B [30]	Amphotericin B is given intravenously over the course of two to six hours	Up to 80% of patients taking amphotericin B develop renal or infusion-related toxicity. Most common side effects: loss of potassium, loss of magnesium, anaphylaxis, fever, and nephrotoxicity	Patients with a history of anaphylactic reactions to amphotericin B should not take the drug. Concomitant steroid use could cause hypokalemia.	Amphotericin B binds to ergosterol in the fungal cell membrane and causes ion channels to form which depolarizes the cell and leads to its death.

## Conclusions

Valley fever, or coccidioidomycosis, is a fungal infection found primarily in the arid environments of the Southwestern United States with an ever-increasing incidence. The study of the disease is instrumental in the prevention of its spread going forward. Advancing our knowledge of the specific human defense mechanisms against *Coccidioides* species would be beneficial in furthering our understanding of the pathogenesis of this disease. One such idea that is not yet fully understood is the primary defense mechanism against *Coccidioides*. It is believed that Th2 lymphocytes are the primary driver for defense given how Th2 deficiency and/or dysfunction is frequently seen with infection; however, further research on the matter to solidify this belief would bolster our understanding of the topic to allow for further advancements against the disease. Research should be performed to uncover the mode in which the virus infects people in an asymptomatic manner, and doing so would allow for future study of the disease to be more thorough.

## References

[1] Akram SM, Koirala J (2023). Coccidioidomycosis. StatPearls [Internet].

[2] Charles-Lozoya S, Ruíz-Zenteno G, Lizcano-Martínez ME, Cobos-Aguilar H, León-Ruíz J, Domínguez-Delgado J (2023). Vertebral coccidioidomycosis with mechanical instability treated solely with antifungals: a case report. Med Mycol Case Rep.

[3] Galgiani JN, Ampel NM, Blair JE, Catanzaro A, Johnson RH, Stevens DA, Williams PL (2005). Coccidioidomycosis. Clin Infect Dis.

[4] Brown J, Benedict K, Park BJ, Thompson GR 3rd (2013). Coccidioidomycosis: epidemiology. Clin Epidemiol.

[5] Smith DJ (2023). Surveillance for Coccidioidomycosis, Histoplasmosis, and Blastomycosis — United States, 2019. MMWR Surveill Summ [Internet.

[6] (2023). Increase in Reported Coccidioidomycosis — United States, 1998-2011. https://www.cdc.gov/mmwr/preview/mmwrhtml/mm6212a1.htm.

[7] Benedict K (2023). Surveillance for Coccidioidomycosis — United States, 2011-2017. MMWR Surveill Summ [Internet.

[8] Ampel NM (2023). The Diagnosis of Coccidioidomycosis. F1000Prime Rep [Internet.

[9] Crum NF (2022). Coccidioidomycosis: a contemporary review. Infect Dis Ther.

[10] (2023). Diagnosis and Testing for Valley Fever (Coccidioidomycosis). https://www.cdc.gov/fungal/diseases/coccidioidomycosis/diagnosis.html.

[11] Williams SL, Chiller T (2022). Update on the epidemiology, diagnosis, and treatment of coccidioidomycosis. J Fungi (Basel).

[12] (2023). About Valley Fever (Coccidioidomycosis). https://www.cdc.gov/fungal/diseases/coccidioidomycosis/definition.html.

[13] (2023). Coccidioidomycosis. https://www.britannica.com/science/coccidioidomycosis.

[14] Blair JE, Logan JL (2001). Coccidioidomycosis in solid organ transplantation. Clin Infect Dis.

[15] Odio CD, Marciano BE, Galgiani JN, Holland SM (2017). Risk factors for disseminated coccidioidomycosis, United States. Emerg Infect Dis.

[16] Nguyen C, Barker BM, Hoover S (2013). Recent advances in our understanding of the environmental, epidemiological, immunological, and clinical dimensions of coccidioidomycosis. Clin Microbiol Rev.

[17] Fernandez JA, Hidalgo MN, Hodzic E, Diab SS, Uzal FA (2018). Pathology of coccidioidomycosis in llamas and alpacas. J Vet Diagn Invest.

[18] Neafsey DE, Barker BM, Sharpton TJ (2010). Population genomic sequencing of Coccidioides fungi reveals recent hybridization and transposon control. Genome Res.

[19] Burt A, Carter DA, Koenig GL, White TJ, Taylor JW (1996). Molecular markers reveal cryptic sex in the human pathogen Coccidioides immitis. Proc Natl Acad Sci U S A.

[20] Fraser JA, Stajich JE, Tarcha EJ, Cole GT, Inglis DO, Sil A, Heitman J (2007). Evolution of the mating type locus: insights gained from the dimorphic primary fungal pathogens Histoplasma capsulatum, Coccidioides immitis, and Coccidioides posadasii. Eukaryot Cell.

[21] Garcia Garcia SC, Salas Alanis JC, Flores MG, Gonzalez Gonzalez SE, Vera Cabrera L, Ocampo Candiani J (2015). Coccidioidomycosis and the skin: a comprehensive review. An Bras Dermatol.

[22] Galgiani JN, Ampel NM, Blair JE (2016). 2016 Infectious Diseases Society of America (IDSA) clinical practice guideline for the treatment of coccidioidomycosis. Clin Infect Dis.

[23] (2023). Valley Fever. https://www.mayoclinic.org/diseases-conditions/valley-fever/diagnosis-treatment/drc-20378765.

[24] (2023). Antifungal Therapy. https://health.ucdavis.edu/valley-fever/about-valley-fever/treatment-antifungal-therapy/index.html.

[25] Sheehan DJ, Hitchcock CA, Sibley CM (1999). Current and emerging azole antifungal agents. Clin Microbiol Rev.

[26] Shibli M, Ghassibi J, Hajal R, O'Sullivan M (2002). Adjunctive corticosteroids therapy in acute respiratory distress syndrome owing to disseminated coccidioidomycosis. Crit Care Med.

[27] Ampel NM (2015). The treatment of coccidioidomycosis. Rev Inst Med Trop Sao Paulo.

[28] Govindarajan A, Bistas KG, Ingold CJ, Aboeed A (2023). Fluconazole. StatPearls [Internet].

[29] Kurn H, Wadhwa R (2023). Itraconazole. StatPearls [Internet].

[30] Noor A, Preuss CV (2023). Amphotericin B. StatPearls [Internet].

